# Methylation of the *SPARC *gene promoter and its clinical implication in pancreatic cancer

**DOI:** 10.1186/1756-9966-29-28

**Published:** 2010-03-26

**Authors:** Jun Gao, Jian Song, Haojie Huang, Zhaoshen Li, Yiqi Du, Jia Cao, Minghui Li, Shunli Lv, Han Lin, Yanfang Gong

**Affiliations:** 1 Department of Gastroenterology, Changhai Hospital, Second Military Medical University, Shanghai 200433, China; 2 Shanghai Biochip Co. Ltd, Shanghai 200433, China

## Abstract

**Background:**

The secreted protein acidic and rich in cysteine (*SPARC*) plays a pivotal role in regulating cell-matrix interactions and tumor angiogenesis, proliferation, and migration. Detection of *SPARC *gene methylation may be useful as a tumorigenesis marker for early detection of pancreatic cancer.

**Methods:**

Methylation of the *SPARC *gene transcriptional regulation region (TRR) was detected using bisulfite-specific (BSP) PCR-based sequencing analysis in 40 cases of pancreatic cancer and the adjacent normal tissues, 6 chronic pancreatitis tissues, and 6 normal pancreatic tissues. BSP cloning-based sequencing analysis was also performed in selected cases. Clinicopathological data from the cancer patients were collected and analyzed.

**Results:**

Analysis of *SPARC *gene TRR methylation showed two hypermethylation wave peak regions: CpG Region 1 (CpG site 1-7) and CpG Region 2 (CpG site 8-12). Pancreatic tissues have shown methylation in both regions with gradual increases from normal, chronic pancreatitis, and adjacent normal tissues to cancerous tissues. However, Methylation of CpG Region 2 was more sensitive than CpG Region 1 in pancreatic tumorigenesis. Furthermore, the methylation level of CpG Region 2 was associated with increased tumor size and exposure to the risk factors (tobacco smoke and alcohol consumption) for developing pancreatic cancer.

**Conclusion:**

Methylation of the *SPARC *gene, specifically CpG Region 2, may be an early event during pancreatic tumorigenesis and should be further evaluated as a tumorigenesis marker for early detection of pancreatic cancer.

## Background

Pancreatic cancer, one of the highly invasive and extremely lethal neoplasms, is the fifth leading cause of cancer death in the United States [1]. Pancreatic cancer mortality almost parallels its incidence, with a 5-year survival rate of less than 4%. Although surgical resection remains the only hope for long-term survival in patients with pancreatic cancer, the majority (~85%) of patients are found to be unresectable at diagnosis due to extensive local invasion and/or metastatic disease [2]. Therefore, early detection of pancreatic cancer is the key for improving survival of patients. Unfortunately, no early-detection markers currently are available for early diagnosis of pancreatic cancer, although many scientists are pursuing pancreatic cancer research and believe that early detection of pancreatic cancer using molecular gene markers may be possible in the future [3,4].

To date, it is clear that many genetic and epigenetic alterations occur during pancreatic tumorigenesis [5]. Among these alterations, methylation of the tumor suppressor gene promoter results in gene silencing [6], which may take place during the very early stages of pancreatic cancer development. Detection of such aberrant DNA methylation of tumor suppressor genes could be used as a diagnostic marker for pancreatic cancer [7]. Thus, defining altered gene expression and understanding the underlying molecular mechanism in pancreatic cancer are urgently needed.

Secreted protein acidic and rich in cysteine (*SPARC*)/osteonectin/BM 40 is a matricellular glycoprotein that is involved in diverse biological processes, including tissue remodeling, wound repair, morphogenesis, cell differentiation, proliferation, migration, and angiogenesis [8-11]. A previous study showed that the *SPARC *gene promoter is aberrantly methylated in primary pancreatic cancer tissue [12]. Gene expression profiling using oligonucleotide microarray and reverse transcription-PCR analyses demonstrated that *SPARC *mRNA was expressed in non-neoplastic pancreatic ductal epithelial cells but not in the majority of pancreatic cancer cell lines [12]. The conditioned medium containing secreted SPARC protein suppressed the growth of pancreatic cancer cells, indicating that silencing of the *SPARC *gene may result in pancreatic cancer development and progression [12].

In the current study, we detected the methylation levels and methylation pattern of the *SPARC *gene transcriptional regulation region (TRR) in normal, adjacent normal, chronic pancreatitis, and pancreatic cancer tissues to assess the altered methylation levels of the SPARC gene to determine if SPARC methylation can be used as a tumorigenesis marker for the early detection of pancreatic cancer.

## Methods

### Cell line and culture

Pancreatic cancer cell line PANC1 was purchased from the American Type Culture Collection (Manassas, VA, USA) and PaTu8988 was a kind gift from Dr. H.P. Elsasser (Phillips University, Marburg, Germany). These cells were grown in Dulbecco's modified Eagle's medium (DMEM) supplemented with 10% fetal bovine serum (both were from Life Technologies Inc., Rockville, MD, USA) and incubated at 37°C in a humidified chamber with 95% air and 5% CO_2_.

### Patient tissue specimens

A tissue and patient's data usage protocol was approved by the Ethics Committee of our institution. Informed written consent was obtained from each patient. Tissue samples from 52 patients were obtained from the Second Military Medical University affiliated Changhai Hospital from August 2006 to December 2007; these samples were from 6 pathologically proven cases of chronic pancreatitis, 6 cases of normal pancreatic tissues, 40 cases of pancreatic cancer (ductal adenocarcinoma type), and corresponding normal tissue from those same 40 patients. The tissue samples were obtained and stored in liquid nitrogen immediately after being resected in the operating room. For pancreatic cancer cases, tumor tissues that contained more than 70% tumor cells and the corresponding adjacent normal tissues without any tumor cell infiltration were selected. In addition, samples of white blood cells (WBCs) were obtained from two healthy volunteers. Clinicopathological data, including gender, age, status of tobacco smoking and alcohol consumption, tumor size, differentiation, lymph node metastasis, and TNM stages, were collected from the electronic medical records of the patients. Tobacco smoking was defined as at least one cigarette per day for no less than 1 year. Alcohol consumption was defined as intake of at least 50 ml of Chinese liquor, 250 ml of wine, or 500 of ml beer at least once a week for a minimum of 1 year. The 6th American Joint Committee on Cancer (AJCC) staging system was used to classify the clinical stage of pancreatic cancer.

### DNA extraction and bisulfite modification of DNA

Genomic DNA from the tissues and cell lines was extracted using the phenol/chloroform method and precipitated with ethanol. One microgram of genomic DNA was subjected to treatment with the EZ DNA Methylation Kit™ (Zymo Research, Orange, CA, USA) according to the manufacturer's instructions. The bisulfite modified DNA was then suspended in 20 μl of deionized water and used immediately or stored at -80°C until use.

### Bisulfite-specific (BSP) PCR and DNA sequencing

The primers used to detect methylation of the *SPARC *gene promoter TRR were designed to specifically amplify bisulfite-converted DNA of *SPARC *TRR. The primers were 5'-ATTTAGTTTAGAGTTTTG-3' (forward) and 5'-ACAAAACTTCCCTCCCTTAC-3' (reverse) and were custom synthesized by Shanghai Sangon (Shanghai, China). Two microliters of the bisulfite modified DNA from each sample were subjected to PCR analysis in a 25 μL volume containing 1 × PCR buffer, 2.0 mmol/L MgCl_2_, 2.5 mmol/L dNTP, 1 mmol/L primer, and EX Taq DNA HS 800 U/L. The reaction mixture was preheated at 95°C for 5 min and amplified using a touch-down PCR program (i.e., 9 cycles of 95°C for 30 s, 59°C for 30 s (next cycle touch-down 0.5°C) and 72°C for 30 s; 42 cycles of 95°C for 30 s, 55°C for 30 s, and 72°C for 30 s; and a final extension of 4 min at 72°C. The PCR products were then subjected to either direct sequencing analysis or cloning into the pMD-18-T vector (TaKaRa, Dalian, China) followed by sequencing analysis (after the cloning, 10-25 clones from each sample were randomly selected for DNA sequencing).

### Sequencing data analysis

Sequencing analysis was performed by Shanghai Invitrogen Biotech Co. Ltd (Shanghai, China). For the data obtained from BSP PCR-based sequencing analysis, the percentage of methylation of each CpG site in a given sample was calculated as the height of the "C" peak divided by the sum of the height of "C" + "T". For the data obtained from BSP cloning-based sequencing analysis, the percentage of methylation of each CpG site in a given sample was calculated as the number of the methylated CpG sites divided by the total observed sequenced clone numbers. The percentage of the region methylation in a given sample was the average of each CpG site in the DNA region.

### Statistical analysis

Statistical analyses were conducted using SPSS version 15.0 (SPSS, Chicago, IL, USA). A one-way ANOVA test was performed to analyze differences in the percentage of the region methylation among pancreatic cancer tissues, adjacent normal pancreatic tissues, chronic pancreatitis tissues, and normal pancreatic tissues. General linear model univariate analysis was performed to determine the correlations of *SPARC *methylation with clinical characteristics of pancreatic cancer. All variables were subsequently analyzed using a stepwise multiple regression to assess their independent contribution to the methylation level, with entry and removal at the 0.05 and 0.1 significance levels, respectively.

## Results

### Methylation of the *SPARC *gene TRR in pancreatic tissues and pancreatic cancer cells

**Figure 1:**
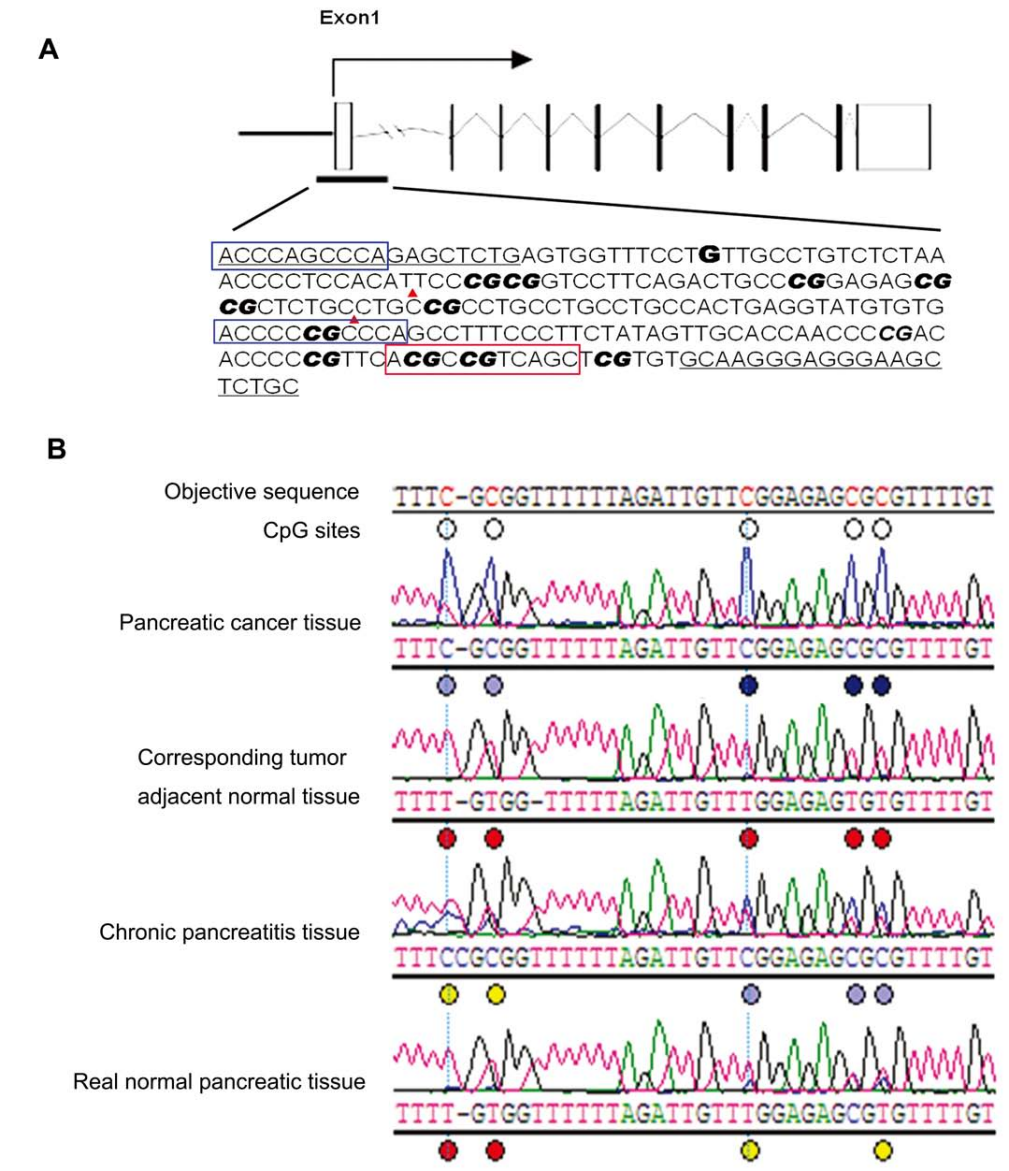
**Detection of SPARC gene TRR methylation**. (A) Illustration of the SPARC gene TRR and topology of the BSP primer. The black bar indicates the analyzed region. The bold "G" indicates the transcriptional start site. The bold italic "CG" indicates the location of 12 CpG island sites. The underlined sequence indicates the primers for BSP. Blue and red rectangles indicate the Sp1 and AP1 binding consensus sequences, respectively. The red triangles indicate the region whose representative sequence analyses were showed in Figure 1B. (B) Representative sequencing data of the SPARC gene TRR in four different groups of pancreatic tissues obtained using BSP PCR-based sequencing analysis. CpG dinucleotides "C" in the objective sequence are shown in red. The red, yellow, green, light blue, and deep blue dots under the analyzed sequence represent different methylation ratios, respectively.

According to the NCBI genome database, we analyzed the *SPARC *gene TRR and found a CpG island around the transcriptional start site (designated as '0') between the upstream -29 bp and downstream +191 bp using the methylation analysis software of Methyl Primer Express v1.0 (ABI). Figure 1A illustrates the structure of the SPARC gene and the topology of the BSP primer, indicating the position of the CpG island containing 12 CpG sites and the BSP primers.

**Figure 2:**
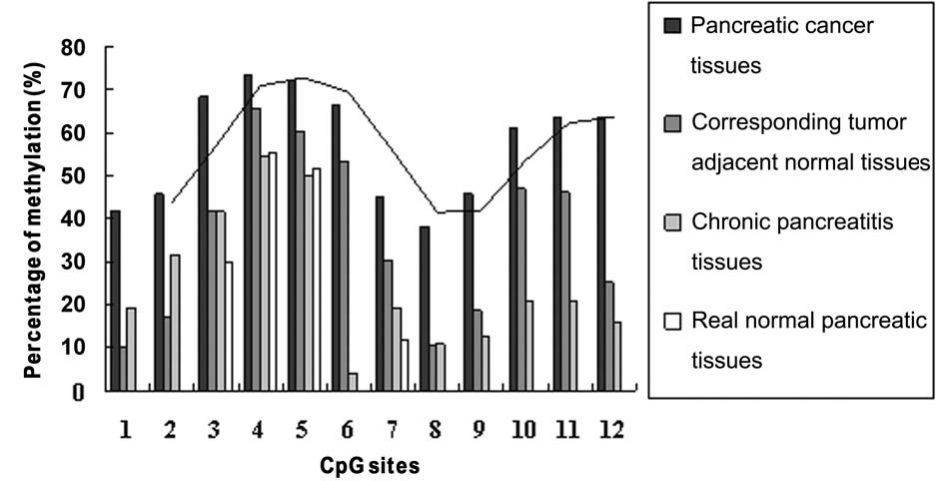
**Methylation pattern of the SPARC gene TRR in pancreatic tissue samples**. The pattern consists of two hypermethylation wave peak regions including CpG region 1 (CpG site 1--7) and CpG region 2 (CpG site 8--12). The curve was fitted to the mean methylation ratios of pancreatic cancer tissues using the MACD (moving average convergence/divergence) method.

**Figure 3:**
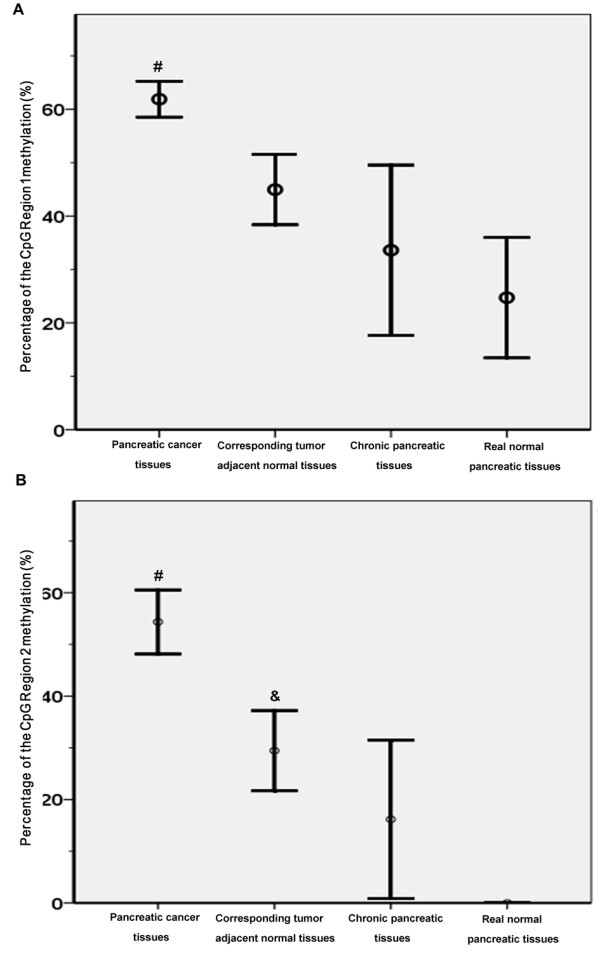
**Methylation level of CpG region 1 (A) and CpG region 2 (B) in the SPARC gene TRR in pancreatic tissues**. All data are reported as means ± 95% CI. #, the pancreatic cancer tissues are compared to the corresponding tumor adjacent normal tissues, chronic pancreatitis tissues, or normal pancreatic tissues, p < 0.05. &, the corresponding tumor adjacent normal tissues are compared to the real normal pancreatic tissues, p < 0.05.

We next performed BSP PCR-based sequencing analysis to assess the methylation status of the *SPARC *gene TRR in four tissue groups: 40 pancreatic cancer samples and their corresponding adjacent normal pancreatic tissues, 6 chronic pancreatitis samples, and 6 real normal pancreatic tissue samples. Figure 1B shows representative BSP PCR-based sequencing analysis results for these four different groups of pancreatic tissues. The methylation pattern of the *SPARC *gene TRR in these four types of pancreatic tissues is shown in Figure 2. According to the curve fitted to the mean percent methylation of pancreatic cancer tissue data by the MACD (moving average convergence/divergence) method, we found two hypermethylation wave peak regions in these CpG islands. The first contained CpG site 1-7 (CpG Region 1) and the second contained CpG sites 8-12 (CpG Region 2). We searched the web site http://www.cbrc.jp/research/db/TFSEARCH.html and found that CpG Region 1 contained two Sp1 sites while CpG Region 2 contained one Ap1 site (Figure 1A). Figure 3 shows the mean percentage of gene methylation and the 95% CI of these two hypermethylation wave peak regions in the four types of pancreatic tissues. Methylation of these two regions appeared to gradually increase from normal, chronic pancreatitis, and adjacent normal to pancreatic cancer tissues. Furthermore, CpG Region 2 was rarely methylated in real normal pancreatic tissues but CpG Region 1 was more frequently methylated in some of normal tissues. In addition, the methylation level of CpG Region 2 in the adjacent normal tissues was significantly increased compared with the normal tissues. These results indicate that methylation of CpG Region 2 may be a more sensitive marker and an early event during pancreatic tumorigenesis.

**Figure 4:**
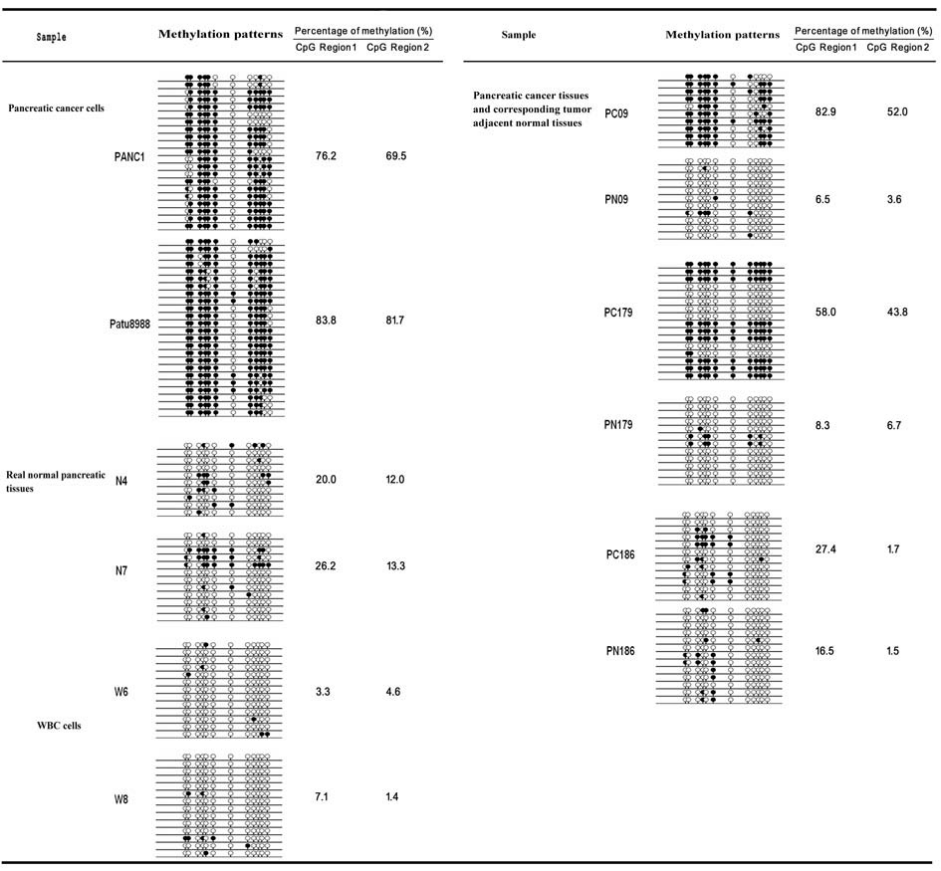
**Methylation status of 12 CpG island sites and the methylation level of CpG Region 1 and CpG Region 2 in the samples determined using BSP cloning-based sequencing analysis**. BSP cloning-based sequencing analysis was performed on real normal pancreatic tissues (N4 and N7), white blood cells (W6 and W8) of two healthy volunteers, pancreatic cancer cell lines (PANC1 and Patu8988), pancreatic cancer tissues (PC09, PC179, and PC186), and the corresponding adjacent normal tissues (PN09, PN179, and PN186). Black dot, methylated; white dot, unmethylated.

To further confirm that hypermethylation of the SPARC gene TRR occurs in pancreatic cancer, we also performed BSP cloning-based sequencing analysis in two pancreatic cancer cell lines (PANC1 and Patu8988), three cases of pancreatic cancer and adjacent normal tissues, two cases of normal pancreatic tissues, and two cases of WBCs from healthy volunteers. Figure 4 shows the methylation pattern of the SPARC gene TRR in these samples. The two pancreatic cancer cell lines and two-thirds of the pancreatic cancer tissues (PC09 and PC179, but not PC186) obviously presented two hypermethylation wave peak regions (CpG Region 1 and CpG Region 2) in the CpG islands compared to the adjacent normal and normal tissues and the WBCs from the healthy volunteers. These data confirmed the results of the BSP PCR-based sequencing analysis.

### Association of *SPARC *gene TRR methylation with clinicopathological parameters in patients with pancreatic cancer

We collected clinicopathological data from the patients and then analyzed the association of *SPARC *gene TRR methylation with clinicopathological parameters in patients with pancreatic cancer. General linear model univariate analysis showed that the percentage of CpG Region 2 methylation was associated with larger tumor size, tobacco smoking, and alcohol consumption (Table 1). Multiple regression analysis also showed that the factors of larger tumor size, tobacco smoking, and alcohol consumption were independent contributors to the percentage of CpG Region 2 methylation (Table 2).

**Table 1:** Correlations of *SPARC *methylation with clinical characteristics of pancreatic cancer

Clinical characteristics	Cases (n = 40)	Methylation ratio (%)					
		
		Region 1	*p*	Region 2	*p*	Total	*p*
Gender							
Male	25	58.6 ± 11.8	0.709	53.6 ± 18.7	0.265	56.5 ± 11.9	0.337
Female	15	59.8 ± 12.1		55.5 ± 22.6		58.0 ± 13.2	
Age (yrs)							
≤ 55	19	58.0 ± 12.0	0.386	52.6 ± 19.1	0.156	55.7 ± 12.1	0.142
> 55	21	60.0 ± 11.7		56.0 ± 21.0		58.3 ± 12.6	
Alcohol							
--	20	58.7 ± 12.9	0.794	46.6 ± 18.2	0.016	53.7 ± 11.2	0.154
+	20	60.0 ± 11.7		62.1 ± 19.1		60.5 ± 12.6	
Smoking							
--	22	58.1 ± 13.7	0.671	47.5 ± 17.5	0.017	53.7 ± 11.9	0.067
+	18	60.2 ± 9.1		62.8 ± 19.1		61.3 ± 11.7	
Tumor size (cm)							
≤ 2	21	55.4 ± 10.5	0.087	46.1 ± 18.8	0.029	51.5 ± 10.1	0.013
> 2	19	63.1 ± 12.0		63.5 ± 17.4		63.3 ± 11.7	
Differentiation							
Moderate	19	59.6 ± 12.2	0.625	53.6 ± 20.4	0.799	57.1 ± 12.4	0.877
Poor	21	58.6 ± 11.6		55.0 ± 20.1		57.1 ± 12.5	
Lymph node metastasis							
--	23	60.4 ± 12.4	0.307	53.7 ± 20.0	0.832	57.6 ± 12.5	0.421
+	17	57.2 ± 10.9		55.2 ± 20.7		56.4 ± 12.3	
pTNM stage							
I+II	21	58.2 ± 12.4	0.444	51.9 ± 20.1	0.867	55.5 ± 12.6	0.543
III+IV	19	60.0 ± 11.2		57.1 ± 20.0		58.8 ± 12.0	

Correlations of *SPARC *methylation with clinical characteristics of pancreatic cancer were determined by general linear model univariate analysis.

**Table 2:** The standardized coefficient beta value of multiple regression analysis

Clinical characteristics	Region 1	Region 2	Whole region
Gender	--	--	--
Age	--	--	--
Alcohol	--	0.341 (*p *= 0.012)	--
Smoking	--	0.336 (*p *= 0.013)	--
Tumor size	0.332 (*p *= 0.036)	0.342 (*p *= 0.013)	0.485 (*p *= 0.002)
Differentiation	--	--	--
Lymph node metastasis	--	--	--
pTNM stage	--	--	--
Adjusted *R*^2^	0.087	0.367	0.215

Clinical characteristics of pancreatic cancer were analyzed using a stepwise multiple regression to assess their independent contribution to the methylation level, with entry and removal at the 0.05 and 0.1 significance levels, respectively.

## Discussion

In the current study, we determined the methylation status of the *SPARC *gene promoter in pancreatic cancer cell lines, pancreatic cancer and corresponding adjacent normal pancreatic tissues, chronic pancreatitis tissues, and real normal pancreatic tissues. Methylation of the *SPARC *gene TRR gradually increased from normal, chronic pancreatitis, and the adjacent normal tissues to pancreatic cancer tissues. The methylation pattern of the *SPARC *gene TRR exhibited two hypermethylation wave peak regions: CpG Region 1 (CpG site 1-7) and CpG Region 2 (CpG site 8-12). CpG Region 2 was rarely methylated in real normal pancreatic tissues but CpG Region 1 was more frequently methylated. In addition, the methylation level of CpG Region 2 in the adjacent normal tissues was significantly increased compared with the real normal tissues. Furthermore, the aberrant hypermethylation of CpG Region 2 was associated with larger tumor size, tobacco smoking, and alcohol consumption. Our results indicated that methylation of CpG Region 2 could be further evaluated as a tumorigenesis marker for the early diagnosis of pancreatic cancer.

It is known that chronic pancreatitis is considered to be a precancerous lesion [13] and that cancer-adjacent tissues experience "the field effect of carcinogenesis," which is evident because they show the same genetic changes as the tumor [14,15]. In this study, we found that CpG Region 2 was hypermethylation in corresponding tumor adjacent normal pancreatic tissues and chronic pancreatitis tissues, and additionally that its hypermethylation correlated with pancreatic cancer risk factors (tobacco smoking and alcohol consumption) [13,16]. These data showed that hypermethyhlation of CpG Region 2 is an early event in pancreatic cancer tumorigenesis.

Brune et al. demonstrated that aberrant methylation of the *SPARC *gene promoter as a marker of sporadic pancreatic adenocarcinoma can also be used to detect familial pancreatic adenocarcinoma [7]. Sato et al. showed that the *SPARC *gene promoter was methylated in pancreatic cancer juice with sensitivity of 90.9% and specificity of 70.4% for pancreatic cancer diagnosis [17]. These studies utilized a conventional MSP method to detect SPARC gene methylation. In the current study, we not only confirmed the published data about methylation of the *SPARC *gene promoter in pancreatic cancer, but we also further revealed the methylation level of the different sites of the CpG island. In particular, our data showed that the methylation pattern of the *SPARC *gene TRR exhibited two hypermethylation wave peak regions. The methylation level of CpG Region 1 was higher in pancreatic cancer tissue than in normal, chronic pancreatitis, and the adjacent normal tissues, but CpG Region 1 of the SPARC gene also was methylated in normal pancreatic tissues. In contrast, CpG Region 2 was only methylated in pancreatic cancer, adjacent normal, and chronic pancreatitis tissues. These data suggest that methylation of CpG Region 2 is a more sensitive marker to detect early alteration in pancreatic cancer.

Aberrant methylation of the *SPARC *gene has been reported in various kinds of tumors, including lung and colorectal cancer, acute myeloid leukemia, multiple myeloma, endometrial cancer, ovarian cancer, cervical cancer, pancreatic cancer, and prostate cancer [18-25]. Infante et al. reported that there were four expression patterns of the SPARC gene in pancreatic cancer tissues: tumor-/stroma- (16%); tumor+/stroma- (17%); tumor-/stroma+ (52%); and tumor+/stroma+ (15%) [26]. Sato et al. reported that *SPARC *mRNA was expressed in non-neoplastic pancreatic ductal epithelial cells (79%) but not in pancreatic cancer cell lines (0/17) or the majority of primary pancreatic cancer tissues (68%) and that methylation of the *SPARC *gene promoter was responsible for gene silencing [12].

The molecular mechanism responsible for methylation of the *SPARC *gene promoter is unknown. Recent studies demonstrated that some environmental factors (such as tobacco smoke) can cause methylation of certain tumor suppressor genes [14,27]. In pancreatic cancer, tobacco smoke can induced k-ras gene mutation and p16 and ppENK gene methylation [28,29]. Our data showed that exposure to risk factors such as tobacco smoke and alcohol use was associated with methylation of CpG Region 2 in the *SPARC *gene promoter in pancreatic cancer tissues. Our data may indicate that these risk factors cause pancreatic cancer development and progression through induction of *SPARC *gene methylation.

The SPARC gene may play a role in suppression of tumorigenesis, including pancreatic cancer. Molecularly, the SPARC protein binds to a number of different extracellular matrix components, such as thrombospondin 1, vitronectin, entactin/nidogen, fibrillar collagens (types I, II, III, and V), and collagen type IV. SPARC has the potential to contribute to the organization of the matrix in connective tissue as well as basement membranes to regulate cell-cell interaction and differentiation to modulate cell growth. However, to date, it remains to be determined whether SPARC is a tumor suppressor gene or an oncogene. It is because both kinds of data were published and available in Pubmed. Particularly, two papers showed that SPARC wasn't expressed in the majority of primary pancreatic cancer tissues (68%~69%)[12,26], whereas another study found high expression of SPARC in almost all tumour tissues [30]. Furthermore, all these three papers reported strong staining of SPARC in fibroblasts and the extracellular matrix. Moreover, Podhajcer et al. [31] reported that SPARC gene expression was associated with good prognosis. In addition, the in vitro experiment showed that the expression of SPARC inhibited growth of cancer cells [12,30], but promoted invasion of pancreatic tumor cells [30]. Another study, however, showed that inhibition of endogenous SPARC enhanced pancreatic cancer cell growth [32]. In our current study, we found that methylation of the SPARC gene is an early event during pancreatic carcinogenesis, which supports the premise that this gene is a tumor suppressor gene. Although we didn't show expression data of SPARC, it is obvious that methylation of gene promoter surely silences the gene expression. Taken altogether, this discrepancy warrants further investigation.

Regulation of gene expression by the de novo methylation is involved in tumorigenesis [33]. De novo methylation is a progressive process rather than a single event and is neither site specific nor completely random but instead is region specific. Recognition and methylation of differentially methylated regions by DNA methyltransferase involves the detection of both nucleosome modification and CpG spacing, giving rise to methylation in a periodic pattern on the DNA [34]. On the other hand, many researchers have found that transcription factors (e.g., c-Myb, c-Myc/Myn, E2F, CREB, Ap1, Ap2, Sp1, and NF-κB) are incapable of binding to methylated DNA of their recognition sequences and that gene transcription therefore is blocked [35,36]. The identification of region-specific methylation patterns in genes may be essential for an accurate assessment of methylation-mediated transcriptional silencing [37]. In this study, two Sp1 and one AP1 sites were identified in the *SPARC *gene TRR and the AP1 site is localized at CpG Region 2 (covering CpG site 10 and CpG site11). However, the biological significance of these SP1 and AP1 sites in the SPARC gene will require further study.

In summary, our current data demonstrated different methylation levels of the *SPARC *gene TRR CpG sites. Methylation of CpG Region 2 was more sensitive than CpG Region 1 in pancreatic tumorigenesis, suggesting that aberrant hypermethylation of CpG Region 2 may be useful as a tumorigenesis marker for early detection of pancreatic cancer. However, this finding needs to be verified in a study with a larger sample size of patients with pancreatic cancer.

## List of abbreviations

SPARC: secreted protein acidic and rich in cysteine; TTR: transcriptional regulation region; BSP: Bisulfite-specific; PANC1: a pancreatic cancer cell line; PaTu8988: a pancreatic cancer cell line; DMEM: Dulbecco's modified Eagle's medium; WBC: white blood cell.

## Competing interests

The authors declare that they have no competing interests.

## Authors' contributions

JG and JS designed the study, wrote the manuscript and performed the statistical analysis. HH participated in its design and participated in the sequence alignment. ZL conceived of the study, and participated in its design. YD and YG collected all the human material and participated DNA extraction and bisulfite modification of DNA. JC, ML, SL and HL performed the methylation detection. JG, JS and HH contributed equally to this work. All authors read and approved the final manuscript.

## Authors' information

Jun Gao, PH.D and MD, Director of the Pancreatic Disease Research Center affiliated to Department of Gastroenterology, Changhai Hospital, Second Military Medical University, Shanghai 200433, China. Manager for the National Scientific Technologic Supporting Project [2006BAI02A12] of "Methods for early pancreatic cancer diagnosis".

Zhaoshen Li, MD, Professor, Maste of Department of Gastroenterology, Changhai Hospital, Second Military Medical University, Shanghai 200433, China. The Chairman of Chinese Society of Digestive Endoscopy. Leader of the National Scientific Technologic Supporting Project [2006BAI02A12] of "Methods for early pancreatic cancer diagnosis".
